# Symptoms of premenstrual dysphoric disorder and cycle phase are associated with enhanced facial emotion detection: An online cross-sectional study

**DOI:** 10.1177/17455057241259176

**Published:** 2024-06-14

**Authors:** Bianca Boboc, Kirsten A Oinonen

**Affiliations:** Department of Psychology, Lakehead University, Thunder Bay, ON, Canada

**Keywords:** disgust, facial emotion detection, facial emotion intensity, hormones, menstrual cycle, premenstrual dysphoric disorder, premenstrual symptoms, sad

## Abstract

**Background:**

Premenstrual dysphoric disorder is a depressive disorder affecting 5%–8% of people with menstrual cycles. Despite evidence that facial emotion detection is altered in depressive disorders, with enhanced detection of negative emotions (negativity bias), minimal research exists on premenstrual dysphoric disorder.

**Objectives:**

The goal of this study was to investigate the effect of premenstrual dysphoric disorder symptoms and the premenstrual phase on accuracy and intensity at detection of facial emotions.

**Design:**

Cross-sectional quasi-experimental design.

**Method:**

The Facial Emotion Detection Task was administered to 72 individuals assigned female at birth with no premenstrual dysphoric disorder (*n* = 30), and provisional PMDD (*n* = 42), based on a retrospective *Diagnostic and Statistical Manual of Mental Disorders—Fifth Edition*-based measure of premenstrual dysphoric disorder. Facial emotion detection was examined both irrespective of menstrual cycle phase, and as a function of premenstrual phase (yes, no). The task used neutral-to-emotional facial expression morphs (15 images/morph). Participants indicated the emotion detected for each image within the progressive intensity morph. For all six basic emotions (sad, angry, fearful, happy, disgust, and surprise), two scores were calculated: accuracy of responses and the intensity within the morph at which the correct emotion was first detected (image number).

**Results:**

Individuals reporting moderate/severe symptoms of premenstrual dysphoric disorder had more accurate and earlier detection of disgust, regardless of cycle phase. In addition, those with provisional premenstrual dysphoric disorder detected sad emotions earlier. A premenstrual dysphoric disorder group × cycle phase interaction also emerged: individuals reporting premenstrual dysphoric disorder symptoms were more accurate at detecting facial emotions during the premenstrual phase compared to the rest of the cycle, with a large effect size for sad emotions.

**Conclusion:**

The findings suggest enhanced facial emotion processing in individuals reporting symptoms of premenstrual dysphoric disorder, particularly for sadness and disgust. However, replication is required with larger samples and prospective designs. This premenstrual dysphoric disorder premenstrual emotion detection advantage suggests an adaptive cognitive mechanism in premenstrual syndrome/premenstrual dysphoric disorder, and challenges stigma surrounding premenstrual experiences.

## Plain Language Summary

Women with Severe Premenstrual Syndrome or Probable Premenstrual Dysphoric Disorder are Better at Identifying Emotional Expressions on People’s Faces, Especially During the Premenstrual Phase

Premenstrual dysphoric disorder is a depressive disorder affecting women where they experience emotional and physical symptoms during the premenstrual phase (i.e. the week before one’s period). It is a severe form of premenstrual syndrome. Research indicates that depression can affect facial emotion recognition. Accurately recognizing other people’s emotions is an important skill that helps us develop social connections and keep ourselves and others safe. Quick recognition of facial emotions allows us to understand and support others, and quickly identify dangerous situations by recognizing other people’s emotional responses. The goal of this study was to examine how premenstrual dysphoric disorder symptoms and the premenstrual phase may affect the ability of women to recognize and identify emotions on other people’s faces. A total of 72 women (42 with premenstrual dysphoric disorder, 30 without premenstrual dysphoric disorder) completed the Facial Emotion Detection Task. This task measured how accurate and early the women were able to detect happiness, sadness, anger, fear, surprise, and disgust in faces. Women with moderate/severe symptoms of premenstrual dysphoric disorder had more accurate and earlier detection of disgust, regardless of where they were in their menstrual cycle. Women with premenstrual dysphoric disorder detected sad emotions earlier. Furthermore, women with premenstrual dysphoric disorder were more accurate at detecting facial emotions when they were tested in the premenstrual phase, and were especially more accurate in detecting sad emotions. The findings suggest that women with premenstrual dysphoric disorder are better at detecting facial emotions and show a premenstrual dysphoric disorder premenstrual emotion detection advantage. This tendency for women with premenstrual dysphoric disorder to better detect emotions in others, particularly when they are in the premenstrual cycle phase, would have benefits. As one of the first reports of a potentially beneficial effect of premenstrual syndrome for women, the findings may help decrease stigma associated with premenstrual dysphoric disorder and premenstrual syndrome. Further research is needed to replicate and extend these findings.

## Introduction

Facial emotion detection (FED) is a component of emotional processing that can guide the perceiver’s affective state and behavior. In the general population, FED has been found to differ across the menstrual cycle,^1–3^ suggesting links with cyclical hormonal change. FED has also been widely studied in relation to depression. Meta-analyses suggest that depression is associated with decreased accuracy of detection across emotions in general, and a negativity bias, whereby individuals with depression detect negative emotions faster and more accurately compared to other emotions.^4–6^ Thus, there is compelling evidence suggesting effects of hormones and mood on FED.

Premenstrual syndrome (PMS) refers to negative physical and emotional symptoms that some people experience during the late-luteal phase and that start to improve at onset of menstruation. The most common emotional symptoms associated with PMS include depressed mood, nervousness, irritability, and tension.^7^ Worldwide it is estimated that 47% of assigned female at birth (AFAB) individuals experience some PMS symptoms.^8^ The experience of relatively mild symptoms is prevalent in the population, however, about 5%–8% of AFAB people experience more severe premenstrual symptoms that cause significant distress and functional impairment.^9^ To recognize these severe symptoms, premenstrual dysphoric disorder (PMDD) was included in the *Diagnostic and Statistical Manual of Mental Disorders*—Fifth Edition (DSM-5) as a depressive disorder.^9^

Unfortunately, many people face barriers in having their PMDD recognized or diagnosed. Furthermore, women’s health concerns are often taken less seriously, and responded to differently, than men’s similar concerns.^10^ Reproductive health issues, including menstruation-related concerns, are particularly prone to dismissal and the belief that symptoms are exaggerated or imagined. In addition, many health care professionals are hesitant to diagnose PMDD, either due to insufficient knowledge,^11^ or the conception that PMDD is not a *real* disorder.^12^ Ultimately, these stigmas neglect AFAB individuals’ health concerns, hindering treatment access, and leading some to take extreme measures^11^ for diagnosis. However, diagnosis offers relief, validation, effective treatment, improved quality of life, and enhanced social connections.^13^ Such findings highlight the importance of research on the features and mechanisms of PMDD.

Although the exact mechanisms in PMS and PMDD are not entirely understood, there is evidence that a hormonal sensitivity likely contributes to symptoms.^14,15^ There are some mixed findings regarding estrogen-, progesterone-, and testosterone-level differences between people with and without PMS/PMDD.^14–16^ However, most recent research suggests that PMS/PMDD is not induced by dysregulated hormone levels, but rather by a hypersensitivity to abrupt hormonal changes.^16,17^ Investigating FED in people with PMS/PMDD may contribute to a better understanding of affective symptoms, unveil potential processing biases, and reveal underlying mechanisms.

Only two studies have examined whether FED differs as a function of the experience of PMS or PMDD. Findings are mixed and slightly different research questions were examined in each. One study found that people with PMDD (but not those without PMDD) were more likely to judge neutral emotions as negative (displayed a negativity bias) during the luteal phase.^18^ Across the menstrual cycle, people with PMDD also made more errors when detecting happy emotions, and were more likely to evaluate happy emotions as negative. Similarly, the second study found that people with PMS were less accurate at detecting expressions of sadness and surprise during the luteal phase compared to the follicular phase.^19^ Both of these studies examined the broader luteal phase as opposed to only the premenstrual phase. A third study found that people with PMDD in the premenstrual phase were more accurate at detecting sad emotions in male faces compared to female faces (i.e. a sex-specific accuracy effect that did not exist in those without PMDD), although they did not directly compare premenstrual performance between people with and without PMDD.^20^ A fourth study looked at FED across the cycle and examined whether PMS symptoms were associated with emotion recognition.^21^ They found that facial emotion recognition was not predicted by PMS symptoms at the time of testing, however, they did not directly examine differences between a PMS versus non-PMS group.^21^ Overall, the findings suggest that people with PMS or PMDD may (a) show a negativity bias (i.e. perceive emotions as more negative), (b) have difficulty discriminating emotional from neutral faces, (c) have difficulty detecting sadness and surprise in the luteal phase, and (d) be better at detecting sadness in male versus female faces. None of the studies to date have directly compared individuals with and without PMS/PMDD in both the premenstrual and non-premenstrual phases or examined the intensity of facial emotions at detection (i.e. how early emotions are detected). Further research is needed.

This study compared FED in people with and without PMDD symptoms as well as between provisional severity groups (no PMDD, mild PMDD, and moderate-severe PMDD) both independent of cycle phase and between the premenstrual and non-premenstrual phases. Accuracy and intensity of detection of the six basic emotions was measured using an intensity morph FED task. Hypothesis 1 was that individuals with provisional PMDD would exhibit a negativity bias, whereby they would detect negative emotions more accurately, and earlier. Hypothesis 2 was that this effect would be strongest during the premenstrual phase.

## Methods

### Participants

The final sample included 72 naturally-cycling (i.e. not taking hormonal contraceptives) AFAB individuals recruited from the university and local community; 30 without PMDD and 42 with provisional PMDD (34 mild and 8 moderate/severe). Participants’ mean age was 22.69 (*SD* = 5.40) years, 66.7% were of European descent, and 72.2% reported high school as their highest achieved education level. In total, 128 participants were recruited and completed the study. Based on a priori exclusion criteria, 56 participants were excluded for having an irregular menstrual cycle (i.e. menses typically occurs more than 2–3 days away from when expected), using hormonal medication within the past 2 months, or consuming alcohol or other cognition-altering substances in the 5 h prior to testing. All participants provided written informed consent and received compensation (course credit and/or gift card draw) for participation.

A minimum sample size of 54 was estimated using GPower 3.1. 36. Sample size analysis was computed considering a medium effect size of 0.25 with α = 0.05 and power = 0.95 (1−ßa).

### Procedure

This study had a cross-sectional between-subjects design and was completed entirely online between January and April 2023. Participants provided written informed consent prior to participating. They then completed a demographic questionnaire, several self-report measures (e.g. a measure of PMDD), and the Facial Emotion Detection Task (FEDT). The demographic questionnaire collected information on demographics (e.g. age, sex, ethnicity), health history (e.g. hormonal contraceptive use, history of psychological disorders and hormonal disorders), substance use, and sleep. It also included questions about the menstrual cycle, including a one-item measure of self-reported menstrual cycle regularity and a one-item measure of frequency and severity of premenstrual symptoms (item 38 in Supplemental Material). The latter item was used as a validity check for group differences. Cycle phase at time of testing was determined by self-report (i.e. “which week of your menstrual cycle are you in”) and by indicating on a calendar the start date of their previous period and the expected start date of their next period. The measures within the demographic questionnaire have been piloted in numerous previous studies in our laboratory.^22–24^ Participants were assigned to the premenstrual group if they were in week four of their cycle, and to the non-premenstrual group if they were in weeks 2 or 3. Individuals currently menstruating (i.e. in week 1) were excluded from the menstrual cycle analyses to limit potential effects of menstrual symptoms (e.g. dysmenorrhea) on the findings.^25^ The STROBE (Strengthening the Reporting of Observational Studies in Epidemiology) guidelines for reporting cross-sectional studies were followed in this report of the study.^26^

### Measures

#### PMDD measure: Screening measure of PMDD symptoms

An 11-item *DSM-5*-based screening tool was used to assess premenstrual symptoms and provisional PMDD.^24^ This scale was developed to assess each criterion in the *DSM*-5 corresponding to PMDD, allowing for both a dimensional and categorical/diagnostic measure of PMS/PMDD. Each item outlines one of the 11 symptoms and asks participants to self-report on the severity of each symptom, the impairment it causes, and the frequency with which it has occurred during the week prior to menstruation over the past year. Evidence of reliability and validity includes high internal consistency (Cronbach’s alpha = .92), and convergent validity (*r* = .70 with another measure of premenstrual symptoms).^24^

Scores can be used to provide a provisional diagnosis of PMDD based on *DSM-5* criteria.^24^ Criterion A was met if participants endorsed experiencing any five symptoms for more than 6 months. Criterion B was met if participants endorsed experiencing at least one of items 1–4 for more than 6 months. Criterion C was met with endorsement of at least one of items 5–11 for more than 6 months. Criterion D was determined by the total scores on the Intensity and Severity scales. If either score was between 0 and 11, Criterion D was not met, indicating that no/minimal PMDD symptoms are present (i.e. no PMDD group). Criterion D was met if Intensity or Severity scores were 12 or greater (i.e. a provisional diagnosis of PMDD). In addition, distress was categorized as mild for scores of 12–32 (i.e. mild PMDD group), and moderate-severe for scores of 33 or higher (i.e. moderate-severe PMDD group).

#### Positive and negative affect schedule

The positive and negative affect schedule (PANAS) was used as to measure current affect.^27^ It contains 20 adjectives describing 10 positive affect states and 10 negative affect states. For each item participants indicate the extent to which they currently feel that way. The scale has been validated and has high internal consistencies (Cronbach’s alpha = .89 for the positive affect subscale and .87 for the negative affect subscale).^27^

#### Facial emotion detection task

The FEDT measured FED as participants viewed and responded to facial emotion images within an intensity morph (a morph from a neutral expression to an emotional expression). Intensity and accuracy of detection of the six basic emotions (fear, sad, angry, disgust, happy, and surprise) was tested.

**Figure 1.**
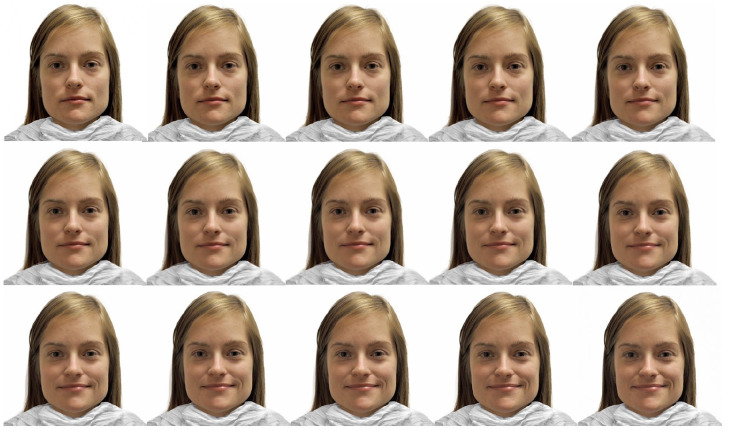
Sample 15-step facial emotion morph. *Note.* A sample 15-step facial emotion morph (from neutral in the upper left image to 100% happy in the lower right image). Note, participants see only one image at a time within the Facial Emotions Detection Task and are asked to report what emotion they perceive. Unmorphed images were retrieved from http://fablab.yale.edu/page/assays-tools and are included here with permission. For more information, see Conley et al.^28^ and Tottenham et al.^29^

The stimuli in the task included images of 24 models expressing neutral and emotional facial expressions, retrieved from the RADIATE face database.^28,29^ A similar morph was piloted in a previous project.^30^ An equal number of male and female; and Black, White, Asian, and Hispanic faces were selected for the morphs, and counterbalanced across the six emotions. The images were morphed using Psychomorph software.^31^ Each model was morphed from neutral to its final target emotion in 15 steps. For each full morph the 15 levels of intensity correlated with an increase of 6.6% intensity of the emotion from each image to the next, such that, the first image within the morph was neutral and 0% emotion, the second image was 6.6% emotion, the third 13.2% emotion, and each subsequent image increased such that the 15th image displayed 100% of the emotion. A sample morph is shown in Figure 1.

The FEDT task was hosted on the website Pavlovia. Participants were informed that all trials would commence with an image of an emotionally neutral face, which over the course of 15 images morphs into one of six detectable emotions, either anger, disgust, fear, happiness, sadness, or surprise. Participants were trained on use of their keyboard to provide emotion responses and were then shown each morph one image at a time. They were instructed to indicate what emotion they perceived in each image. Once participants indicated the emotion they perceived, only then was the next image in the morph presented, and participants were required to respond to each image in the morph, regardless of whether they responded correctly. Invalid trials were identified as those with incomplete responses, or those in which participants did not respond validly (e.g. persisted with one response the entire trial). All trials were manually inspected for invalidity and single key mistakes by two raters. Supplemental materials providing further details on determining trial validity can be found on the journal website.

#### Intensity measure

Intensity level of detection is represented by the image number at which participants reported the correct emotion for each trial. The Image Number at Detection score equaled the image number within the morph at which participants reported the correct emotion, ranging from 2 to 15. On trials in which participants never detected the correct final emotion, they were assigned an intensity level of 17, which corresponds to two units larger than the highest possible correct image number. This value was determined such that scores would be sensitive to error trials, while maintaining normal distribution of the variable. The Image Number at Detection for each of the six emotion types was computed as the mean Image Number at Detection across the four trials of that emotion (i.e. six scores). Lower scores represent earlier detection and indicate better performance.

#### Accuracy measure

Accuracy scores were calculated to reflect Percentage of Incorrect Responses participants made for each emotion. The Percentage of Incorrect Responses for each emotion was computed as the number of incorrect responses divided by the total number of possible responses across trials for that emotion type. The total number of possible responses depended on the number of valid trials. If the participant had no invalid trials, their total possible responses was 60 per emotion (i.e. 4 trials × 15 possible responses per trial), and the number decreased by 15 for every invalid trial. For example:

$$\mathrm{Happy}\;\mathrm{Percentage}\;\mathrm{of}\;\mathrm{Incorrect}\;\mathrm{Responses}=\frac{\#\mathrm{Disgust},\;\mathrm{Fear},\;\mathrm{Sad},\;\mathrm{Angry},\;\mathrm{Surprise}\;\mathrm{Responses}}{15\;(4-\#\mathrm{Invalid}\;\mathrm{Happy}\;\mathrm{Trials})}$$

Lower scores represent higher accuracy and indicate better performance.

## Statistical analysis

To test hypothesis 1, FEDT scores of three groups (no PMDD, mild PMDD, and moderate-severe PMDD) were compared regardless of cycle phase. Two separate three-group multivariate analyses of covariance (MANCOVAs) were conducted with the following dependent variables (DVs): (a) Image Number at Detection for fearful, sad, and angry emotions (i.e. intensity for negative emotions) and (b) Percentage of Incorrect Responses for fearful, sad, and angry emotions (i.e. accuracy for negative emotions). Meaningful MANCOVA results (Pillai’s trace *F-*statistic with α < .05 or partial eta-squared (η)^2^ > .10) were followed-up with univariate analyses of covariance (ANCOVAs) and pairwise comparisons when significant. Effect sizes were defined as small (> .01), medium (> .06), and large (> .14).^32^ Exploratory MANCOVAs (and follow-up ANCOVAs) were run to examine detection differences between the three groups across all six emotions.

To test hypothesis 2, FEDT scores of the no PMDD group and the PMDD group were compared as a function of cycle phase (non-premenstrual and premenstrual). Two separate between-subjects 2 × 2 MANCOVAs were conducted to examine the interaction effect of PMDD group and the premenstrual phase (weeks 2–3 versus week 4), on (a) Image Number at Detection for fearful, sad, and angry emotions and (b) Percentage of Incorrect Responses for fearful, sad, and angry emotions. Meaningful MANCOVA results (defined above) were followed-up with univariate ANCOVAs and pairwise comparisons as appropriate. Exploratory 2 × 2 MANCOVAs (with follow-ups) also examined the effects of PMDD group (no PMDD versus PMDD) and the premenstrual phase (weeks 2–3 versus week 4), on detection across all six emotions.

The following variables were considered as potential covariates: age, body mass index (BMI), typical alcohol use, typical tetrahydrocannabinol (THC) use, hours of sleep last night, typical hours of sleep, ethnicity, education, typing skills, MDD diagnosis, attention-deficit/hyperactivity disorder (ADHD) diagnosis, caffeine withdrawal, and nicotine withdrawal.

## Results

### Validity of PMDD groups and group equivalency

The moderate-severe PMDD group reported more PMS symptoms than the combined no and mild PMDD groups (*p* = .028). Furthermore, the no PMDD group reported less frequent/severe PMS symptoms (on a separate one-item measure) than the combined mild and moderate-severe PMDD groups (*p* = .004), supporting the validity of groups (i.e. no PMDD versus PMDD). PMDD groups also differed in the level of their current positive affect (*F* (2, 69) = 4.090, *p* = .021), with positive affect scores being highest within the no PMDD group (*M* = 23.16, *SD* = 9.00), followed by the mild PMDD group (*M* = 19.45, *SD* = 7.29), and lowest in the moderate-severe PMDD group (*M* = 15.00, *SD* = 2.78). Negative affect also differed across the groups (*F* (2, 69) = 3.588, *p* = .033) and followed the expected pattern (no PMDD (*M* = 14.57, *SD* = 5.98) < mild PMDD (*M* = 17.64, *SD* = 6.22) < moderate-severe PMDD (*M* = 20.25, *SD* = 5.99)). PMDD is associated with higher negative affect and lower positive affect, so these group differences were expected.^33^

The no, mild, and moderate-severe PMDD groups did not differ significantly on any of the covariates assessed (all *p* > .05), except for typical alcohol use over the past 6 months (*F* (2, 69) = 4.087, *p* = .021), with typical alcohol use scores (a composite of consumption frequency and amount over the past 6 months) being lowest within the no PMDD group (no PMDD (*M* = 3.50, *SD* = 2.45), mild PMDD (*M* = 5.26, *SD* = 3.04), moderate-severe PMDD (*M* = 5.88, *SD* = 3.04)). As participants with higher typical alcohol use had earlier surprise detection (*r*(72) = –.241, *p* = .041), and other evidence suggests alcohol use is associated with impaired FED,^34^ typical alcohol use was included as a covariate in all analyses. While PMDD groups did not differ in the hours of sleep they had the night before testing, other research suggests that a previous night’s sleep can affect FED,^35^ and sleep was associated with earlier detection of surprise (surprise Image Number at Detection, *r*(160) = –.221, *p* = .005) and more errors when detecting disgust (disgust Percentage of Incorrect Responses, *r*(160) = .174, *p* = .037). Thus, hours of sleep last night was also included as a covariate in all analyses.

## Intensity

Table 1 contains the unadjusted means and *SD*s of Image Number at Detection scores. The three-group (no PMDD, mild PMDD, moderate-severe PMDD) MANCOVAs comparing groups on Image Number at Detection across all negative emotions (*F*(6, 71) = 1.412, *p* = .215, η^2^ = .061) and across all six emotions were not significant (*F*(12, 71) = 1.740, *p* = .066, η^2^ = .144). However, a non-significant trend with a moderate-large effect size did present across all emotions, so follow-up ANCOVAs were performed (see Table 1).

**Table 1.** Unadjusted means and *SD*s for Image Number at Detection per emotion for no, mild, and moderate-severe provisional premenstrual dysphoric disorder (PMDD) groups.

Emotion	Mean (*SD*) of image number at detection			Follow-up ANCOVAs (*df* = 2,66)		
	No PMDD (*n* = 30)	Mild PMDD (*n* = 34)	Moderate-severe PMDD (*n* = 8)	*F*	*p*	η^2^
Fear	10.67 (2.36)	9.89 (2.41)	10.53 (2.20)	0.869	.424	.026
Sad^t^	9.17 (2.04)	8.65 (1.68)	8.31 (1.28)	2.606	.081	.073
Angry	10.06 (1.89)	9.54 (2.21)	10.53 (1.75)	0.858	.429	.025
Disgust*	10.21 (1.75)	11.32 (2.13)^y^	9.38 (1.59)^y^	3.969	.024	.107
Happy	6.38 (1.64)	6.34 (1.81)	6.78 (1.40)	0.229	.796	.007
Surprise	7.16 (1.73)	6.66 (1.98)	7.03 (1.24)	0.648	.526	.019

*Note.* Lower mean scores indicate earlier (i.e. lower intensity of emotion) detection. The three variables used in the negative emotion MANCOVA are shown at the top of the table. The full table shows the six dependent variables used within the overall emotion MANCOVA. The means are unadjusted for covariates, but all analyses controlled for hours of sleep last night and typical alcohol consumption. ^x^Group differences between the indicated group and the other two groups. ^y^Group differences between the two indicated groups.

t *p* *<* .10. *p < .05. **p < .01. ***p < .001.

**Figure 2.**
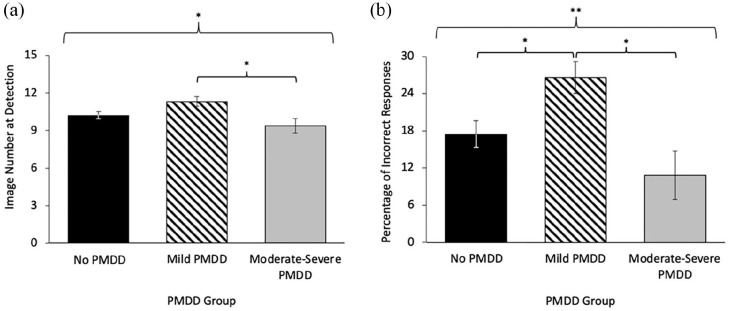
Disgust detection: (a) Image Number at Detection and (b) Percentage of Incorrect Responses as a function of provisional premenstrual dysphoric disorder (PMDD) groups (no, mild, and moderate-severe). *Note.* Women with moderate-severe provisional PMDD detected disgust earlier and more accurately. (a) Lower scores indicate earlier (i.e. requiring lower intensity of emotion) detection. There was a univariate effect for group in Image Number at Detection for disgust, *F*(12, 71) = 3.969, *p* = .024, η^2^ = .107. The moderate-severe PMDD group detected disgust emotions earlier than the mild PMDD group (*p* *=* .038). (b) Lower scores indicate a lower percentage of errors. There was a univariate effect for group in Percentage of Incorrect Responses for disgust, *F*(12, 71) = 5.971, *p* = .004, η^2^ = .153. The no PMDD group (*p* = .043), and the moderate-severe PMDD group (*p* *=* .014), made fewer incorrect responses than the mild PMDD group. All analyses controlled for hours of sleep last night and typical alcohol use and adjusted means are presented. Error bars represent standard error. ^t^*p* *<* .10. *p < .05. **p < .01. ***p < .001.

ANCOVAs revealed that PMDD groups differed in their Image Number at Detection for disgust emotions (*p* = .024, η^2^ = .107), and a non-significant trend emerged for sad emotions (*p* = .081, η^2^ = .073). Pairwise comparisons determined that the moderate-severe PMDD group detected disgust significantly earlier than the mild PMDD group (*p* *=* .038). Figure 2(a) displays the group differences in intensity for disgust. When an exploratory analysis was re-run with menstrual cycle phase included as a covariate, the results did not change.

## Accuracy

Table 2 contains the unadjusted means and *SD*s of Percentage of Incorrect Responses scores. The three-group MANCOVA examining Percentage of Incorrect Responses across the negative emotions was non-significant, *F*(6, 71) = 1.082, *p* = .376, η^2^ = .048. The three-group MANCOVA testing Percentage of Incorrect Responses across all emotions, was also not significant, *F*(12, 71) = 1.376, *p* = .186, η^2^ = .117. Given the moderate-large effect size, follow-up ANCOVAs for intensity at detection for each of the emotions were performed (see Table 2—right panel).

**Table 2.** Unadjusted means and *SD*s for Percentage of Incorrect Responses per emotion for no, mild, and moderate-severe provisional premenstrual dysphoric disorder (PMDD) groups.

Emotion	Mean (*SD*) Image Number at Detection			Follow-up ANCOVAs (df = 2,66)		
	No PMDD (*n* = 30)	Mild PMDD (*n* = 34)	Moderate-severe PMDD (*n* = 8)	*F*	*p*	η^2^
Fear	16.78 (12.98)	14.4 (11.52)	12.71 (11.98)	0.772	.466	.023
Sad^t^	1.59 (3.66)	4.36 (6.15)	0.63 (1.77)	2.446	.094	.069
Angry	6.11 (8.83)	6.76 (11.11)	5.97 (9.35)	0.124	.883	.004
Disgust**	17.44 (11.80)	26.59 (14.39)^x^	10.83 (10.98)	5.971	.004	.153
Happy	1.33 (4.68)	0.34 (0.99)	0.63 (1.77)	0.545	.582	.016
Surprise	4.94 (7.85)	5.74 (7.70)	0.42 (1.18)	1.445	.243	.042

*Note.* Lower scores indicate a lower percentage of errors when detecting trials with the identified emotion. The three variables pertaining to the negative emotion MANCOVA are shown at the top of the table. The full table shows the dependent variables used within the overall emotion MANCOVA. The data are unadjusted for covariates, but all analyses controlled for hours of sleep last night and typical alcohol use. ^x^Group differences between the indicated group and the other two groups. ^y^Group differences between the two indicated groups.

t *p* *<* .10. *p < .05. **p < .01. ***p < .001.

The ANCOVAs revealed that PMDD groups differed in their Percentage of Incorrect Responses for disgust emotions (*p* = .004, η^2^ = .153), and a non-significant trend emerged for sad emotions (*p* = .094, η^2^ = .069). The mild PMDD group made more incorrect responses with disgust emotions than the no PMDD group (*p* = .043), and the moderate-severe PMDD group (*p* *=* .014). Figure 2(b) displays the group differences in accuracy for disgust. When an exploratory analysis was re-run with menstrual cycle phase included as a covariate, the results did not change.

**Figure 3.**
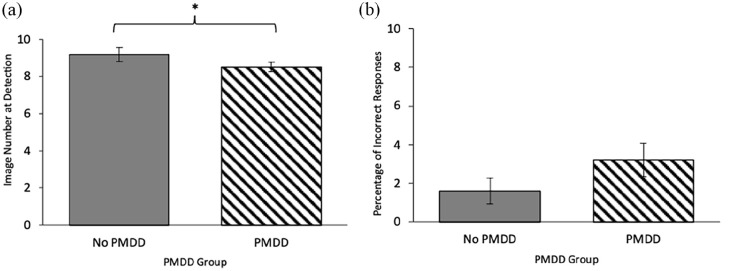
Sad detection: (a) Image Number at Detection and (b) Percentage of Incorrect Responses as a function of provisional premenstrual dysphoric disorder (PMDD) groups (no/yes). *Note.* Women with provisional PMDD detected sad earlier. (a) Lower scores indicate earlier (i.e. requiring lower intensity of emotion) detection. There was a univariate effect for group in Image Number at Detection for disgust, *F*(1, 67) 4.803, *p* = .032, η^2^ = .067. (b) Lower scores indicate a lower percentage of errors. There were no significant group differences. All analyses controlled for hours of sleep last night and typical alcohol use and adjusted means are presented. Error bars represent standard error. ^t^*p* < 10. *p < .05. **p < .01. ***p < .001.

Given the small sample size in the moderate-severe PMDD group (*n* = 8), the ANCOVAs for Image Number at Detection and Percentage of Incorrect Responses for disgust and sad emotions were re-run using two larger groups: no PMDD and PMDD. Table 3 contains the unadjusted means and *SD*s of Image Number at Detection and Percentage of Incorrect Responses, and the ANCOVA results. Image Number at Detection for disgust was no longer significantly different between the no PMDD and the PMDD groups. However, a non-significant trend suggested the PMDD group had a higher Percentage of Incorrect Responses for disgust emotions (less accurate), than the no PMDD group (*p* = .089, η^2^ = .043). Also, the PMDD group detected sad emotions earlier than the no PMDD group (*p* = .032, η^2^ = .067), while Percentage of Incorrect Responses for sad did not differ between the groups. Figure 3 displays the group differences in intensity and accuracy for sad emotions. This suggests sad emotions are detected earlier in people reporting provisional PMDD than those without PMDD.

**Table 3.** Unadjusted means (*SD*s) and ANCOVA results for Image Number at Detection and Percentage of Incorrect Responses per emotion for the provisional premenstrual dysphoric disorder (PMDD) groups (no/yes).

Emotion	No PMDD (*n* = 30)	PMDD (*n* = 42)	ANCOVA results			
	Image Number at Detection		*df*	*F*	*p*	η^2^
Sad*	9.17 (2.04)	8.50 (1.53)	1, 67	4.803	.032	.067
Disgust	10.21 (1.75)	10.89 (2.17)	1, 67	1.254	.267	.018
	Percentage of Incorrect Responses		*df*	*F*	*p*	η^2^
Sad	1.59 (3.66)	3.21 (5.07)	1, 67	1.490	.227	.022
Disgust^t^	17.44 (11.80)	23.59 (15.23)	1, 67	2.985	.089	.043

*Note.* Lower Image Number at Detection scores indicates earlier (i.e. requiring lower intensity of emotion) detection. Lower Percentage of Incorrect Responses scores indicates a lower percentage of errors when detecting trials with the identified emotion. The data are unadjusted for covariates, but all analyses controlled for hours of sleep last night and typical alcohol use.

t *p* *<* .10. *p < .05. **p < .01. ***p < .001.

## Intensity and accuracy within the premenstrual phase

As sample sizes were small for subgroups in the premenstrual phase, analyses were considered preliminary and were undertaken to inform future research on FED during the premenstrual phase. Table 4 contains the unadjusted means and SDs of Image Number at Detection scores pertaining to the 2 (PMDD group: yes, no) × 2 (cycle phase: premenstrual, non-premenstrual) MANCOVAs. There were no significant group × phase interaction effects across: (a) all negative emotions, *F*(3, 42) = 0.517, *p* = .673, η^2^ = .036, or (b) all emotions, *F*(6, 39) = 0.692, *p* = .657, η^2^ = .096. These findings suggest that PMDD groups did not differ in their Image Number at Detection (i.e. intensity) for negative or overall emotions based on whether individuals were in the premenstrual phase.

**Table 4.** Unadjusted means and *SD*s of Image Number at Detection per emotion for the provisional premenstrual dysphoric disorder (PMDD) groups (no/yes) as a function of cycle phase.

Emotion	Mean (*SD*) Image Number at Detection			
	Non-premenstrual phase		Premenstrual phase	
	No PMDD (*n* = 17)	PMDD (*n* = 18)	No PMDD (*n* = 5)	PMDD (*n* = 10)
Fear	10.85 (2.91)	9.63 (2.73)	9.90 (1.04)	10.15 (1.94)
Sad	9.25 (2.33)	8.46 (1.58)	8.75 (1.98)	8.78 (1.59)
Angry	10.51 (1.86)	9.10 (1.92)	9.45 (2.00)	9.60 (2.18)
Disgust	10.47 (1.64)	11.14 (2.58)	10.35 (1.97)	10.13 (1.80)
Happy	6.35 (1.86)	6.31 (2.07)	5.95 (1.44)	5.83 (1.39)
Surprise	7.53 (1.88)	6.78 (2.14)	7.10 (2.05)	6.13 (1.47)

*Note.* Lower scores indicate earlier (i.e. lower intensity of emotion) detection. The non-premenstrual phase encompasses those in weeks 2–3 of the menstrual cycle at the time of testing, and the premenstrual phase encompasses those in week 4. The three variables used in the negative emotion MANCOVA are shown at the top of the table. The full table shows the six dependent variables used within the overall emotion MANCOVA. Significance values reflect the results of follow-up ANCOVAs. The data are unadjusted for covariates, but all analyses controlled for hours of sleep last night and typical alcohol use.

t *p* *<* .10. *p < .05. **p < .01. ***p < .001.

Table 5 contains the unadjusted means and SDs for Percentage of Incorrect Responses scores pertaining to the 2 (PMDD group) × 2 (cycle phase) MANCOVAs. The group × phase interaction effects were significant across both (a) all negative emotions, *F*(3, 42) = 5.966, *p* = .002, η^2^ = .299 and (b) all emotions, *F*(6, 39) = 3.492, *p* = .007, η^2^ = .349. To follow-up on the interaction for all emotions, a 2 (PMDD group) × 2 (cycle phase) ANCOVA examined total Percentage of Incorrect Responses across all emotions (total Percentage Incorrect Responses). There was a significant group × phase interaction, *F*(5, 44) = 5.029, *p* = .030, η^2^ = .103. Follow-up pairwise comparisons between the four groups indicated that individuals reporting provisional PMDD were more accurate across all facial emotions when they were in the premenstrual phase (*M* = 5.86, *SD* = 2.94) compared to the rest of the cycle (*M* = 10.01, *SD* = 5.52; *p* = .034), but did not differ from the premenstrual no PMDD group (*M* = 10.17, *SD* = 6.46; *p* = .101) or non-premenstrual no PMDD group (*M* = 7.30, *SD* = 4.37: *p* > .05).

**Table 5.** Unadjusted means (*SD*s) for Percentage of Incorrect Responses per emotion for provisional premenstrual dysphoric disorder (PMDD) groups (no/yes) as a function of cycle phase.

Emotion	Means (*SD*) of Percentage of Incorrect Responses			
	Non-premenstrual phase		Premenstrual phase	
	No PMDD (*n* = 17)	PMDD (*n* = 18)	No PMDD (*n* = 5)	PMDD (*n* = 10)
Fear	15.78 (13.91)	15.15 (14.29)	20.00 (13.99)	14.67 (8.45)
Sad***	0.36 (1.13)^x,y^	5.09 (5.61)^x,w^	6.67 (6.12)^z,y^	0.50 (1.58)^z,w^
Angry	5.20 (7.52)	5.93 (11.00)	8.00 (8.37)	3.06 (4.88)
Disgust	18.04 (10.04)	27.59 (16.85)^y^	16.67 (17.00)	14.17 (10.04)^y^
Happy	0.20 (0.81)	0.19 (0.54)	0.00 (0.00)	0.50 (1.58)
Surprise*	3.73 (6.42)	6.20 (7.64)	9.67 (10.95)	2.33 (3.26)

*Note.* Lower scores indicate a lower percentage of errors when detecting trials with the identified emotion. The non-premenstrual phase encompasses those in menstrual cycle weeks 2–3 at the time of testing, and the premenstrual phase encompasses those in week 4. The three variables pertaining to the negative emotion MANCOVA are shown at the top of the table. The full table shows the dependent variables used within the MANCOVA on all emotions. Significance values reflect the results of follow-up ANCOVAs. The data are unadjusted for covariates, but all analyses controlled for hours of sleep last night and typical alcohol use. ^w,x,y,z^Group differences between the two indicated groups.

t *p* *<* .10. *p < .05. **p < .01. ***p < .001.

**Figure 4.**
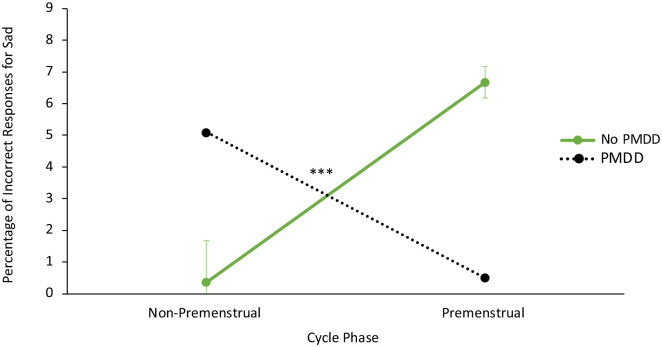
Interaction between provisional premenstrual dysphoric disorder (PMDD) group (no/yes) and cycle phase (premenstrual, non-premenstrual) on Percentage of Incorrect Responses for sad emotions. *Note*. Lower scores indicate a lower percentage of errors when detecting trials with sad emotions. Non-premenstrual phase = weeks 2–3 at the time of testing. Premenstrual phase = week 4 at the time of testing. There was a significant PMDD group × phase interaction effect for Percentage of Incorrect Responses for sad emotions, *F*(1, 63) = 17.768, *p* < .001, η^2^ = .288. Women with PMDD who were tested during the premenstrual phase made fewer errors than PMDD women during the non-premenstrual phase (*p* = .004) and than women without PMDD during the premenstrual phase (*p* = .005). Error bars represent standard errors. ^t^*p* < .10. *p < .05. **p < .01. ***p < .001.

Table 6 contains all ANCOVA results on individual emotions, as a follow-up to the above MANCOVA. Figure 3 illustrates the group differences in Percentage of Incorrect Responses. ANCOVAs examining Percentage of Incorrect Responses for individual emotions revealed significant group × phase interactions for the detection of sad (*p* < .001, η^2^ = .288), and surprise (*p* = .030, η^2^ = .102) emotions. The premenstrual PMDD group was more accurate at detecting sad emotions than the premenstrual no PMDD group (*p* = .005) and the non-premenstrual PMDD group (*p* = .004). Conversely, the non-premenstrual no PMDD group was more accurate at detecting sad emotions than the non-premenstrual PMDD group (*p* = .005), and the premenstrual no PMDD group (*p* = .005). See Figure 4 for an illustration of this interaction. The same relationship presented for surprise emotions, although pairwise comparisons were not significant (all *p* > .05). Finally, while the relevant ANCOVA was not significant, it is noteworthy that the premenstrual PMDD group was significantly more accurate at detecting disgust emotions compared to the non-premenstrual PMDD group (*p* = .017) (see Table 5). Overall, individuals reporting provisional PMDD were more accurate in detecting sad and disgust facial emotions when they were in the premenstrual phase compared to the rest of the cycle.

**Table 6.** ANCOVA interaction results for Percentage of Incorrect Responses per emotion: provisional premenstrual dysphoric disorder (PMDD) groups (no/yes) × cycle phase (non-premenstrual/premenstrual).

Percentage of incorrect responses				
Emotion	*df*	*F*	*p*	η^2^
Fear	1, 63	0.292	.592	.007
Sad***	1, 63	17.768	< .001	.288
Angry	1, 63	0.996	.324	.022
Disgust	1, 63	1.847	.181	.040
Happy	1, 63	0.739	.395	.017
Surprise*	1, 63	5.004	.030	.102

*Note.* Results of the follow-up univariate ANCOVAs testing the interaction effect of PMDD group (no PMDD versus PMDD) and cycle phase (weeks 2–3 versus week 4 (premenstrual phase)) in Percentage of Incorrect Responses for individual emotions. All analyses controlled for hours of sleep last night and typical alcohol consumption.

t *p* *<* .10. *p < .05. **p < .01. ***p < .001.

## Discussion

In line with hypothesis 1, participants reporting provisional PMDD detected sad emotions earlier compared to people without PMDD. Consistent with hypothesis 2, an overall large effect size group × phase interaction indicated that people reporting provisional PMDD were more accurate in detecting negative emotions (when all emotions were examined together) when they were in the premenstrual phase versus other parts of the cycle. This was particularly true for the detection of sad emotions.

### PMDD associated with more accurate emotion detection during the premenstrual phase

The large effect size group × phase interactions indicated that individuals reporting PMDD symptoms in the premenstrual phase may be most accurate at detecting (a) negative emotions, (b) emotions in general, and (c) sad emotions specifically. The premenstrual PMDD group was significantly more accurate at detecting sad emotions than the premenstrual no PMDD group and the non-premenstrual PMDD group. A similar significant interaction for surprise emotions also presented with a medium-large effect size. A final significant group difference indicated that the PMDD group was more accurate in detecting disgust within the premenstrual than the non-premenstrual phase.

Only three previous studies have examined FED in PMS/PMDD groups, and only two examined PMDD–cycle-phase interactions. In one study, people with PMDD were more likely to rate neutral emotions as sad in the luteal phase.^18^ Taken together, our results may suggest that people with PMDD have a tendency or bias toward the perception of sadness during the luteal/premenstrual phase (i.e. more accurate perception of sadness in our study and a greater tendency to view neutral faces as sad in the previous study).^18^ However, another study looking at the broader luteal phase (i.e. not specific to the premenstrual phase) used a within-subjects design and found people with PMS were less accurate at detecting sad emotions during the luteal phase compared to the follicular phase, and there were no differences between individuals with and without PMS at any point.^19^ One final study found that people reporting PMDD symptoms were better at detecting sadness in male compared to female faces.^20^ It should be noted that our study defined the premenstrual phase as week 4 of the menstrual cycle, while the non-premenstrual phase was defined as weeks 2–3. Our approach also differs from past studies, which have often looked at the full luteal, instead of the late-luteal phase or which have not compared people with and without PMDD in both the premenstrual and non-premenstrual phases. Furthermore, our study was the first to specifically compare individuals with and without provisional PMDD in the late-luteal/premenstrual phase compared to the rest of the cycle, and our findings are consistent with two previous studies in suggesting that there is something unique about how people with PMDD process sad facial emotions in the luteal phase.

The present findings suggest that people reporting provisional PMDD may experience a tendency toward enhanced facial emotion processing during their premenstrual phase (i.e. greater accuracy in detecting emotions). This provides evidence of a cognitive-perceptual difference between individuals with and without PMDD. The finding also supports the classification of PMDD as a distinct condition that differs from major depressive disorder (MDD) where one would not expect a cyclical effect for FED. This premenstrual dysphoric disorder premenstrual emotion detection advantage (PMDD-PEDA) may represent an adaptive mechanism within PMDD. While enhanced detection of negative emotions may negatively impact the mood of the perceiver,^4,6^ the enhanced processing of facial emotions in general could allow those with PMDD to harness stronger social/parenting relationships and connection in general and during the premenstrual phase. Earlier or more accurate recognition of some emotions (i.e. disgust, anger, or fear) could also provide a health and safety advantage by facilitating avoidance of spoiled foods, or escape from dangerous situations. This advantage may partially offset any adverse emotional and physical symptoms experienced with PMDD.

### PMDD associated with earlier detection of sad emotions

When individuals with and without provisional PMDD were compared on FED independent of cycle phase, the PMDD group was earlier to detect sad emotions than the no PMDD group, with a medium effect size. This suggests that enhanced detection of sad emotions among individuals reporting provisional PMDD may also exist across the entire cycle (in addition to a premenstrual peak) and supports the notion of a general negativity bias/advantage within PMDD.

PMDD is characterized as a depressive disorder, and mood lability is one of the essential features of the disorder.^36^ There is strong evidence suggesting that individuals with MDD are more likely to categorize neutral expressions as negative and exhibit attentional biases toward negative emotions, which is commonly referred to as a negativity bias.^4,6^ Within depression, this bias is hypothesized to be a part of a bidirectional relationship between negative affect and emotional processing, contributing to the development and maintenance of depression. Enhanced detection of sad emotions by people reporting provisional PMDD could be indicative of a negativity bias within PMDD, which may be explained by a shared mechanism contributing to PMDD and other depressive disorders, such as MDD. Similar to the negativity bias within depression, this negativity bias during the premenstrual phase and across the cycle in individuals with PMDD may prime individuals to exhibit a hypervigilance to negative emotional stimuli. This can cause one to attend to negative stimuli, and perceive other kinds of emotional stimuli as more negative, which can reinforce the negative affective state that people with PMDD report during the premenstrual phase.^4^

While the current study is only the second^18^ to find evidence of enhanced negative facial emotion processing in PMDD, other research indicates this tendency persists when producing and processing other kinds of emotional stimuli as well. One study found people with PMS symptoms produced more sad facial expressions when viewing emotionally charged stimuli during the luteal phase.^37^ Another study asked people about their experience of negative, positive, and neutral life events over different timelines (ranging from the past month to the past 3 years).^38^ When tested in the late-follicular phase, PMS symptoms were positively associated with reporting more negative life events and less positive life events. Similarly, when led to believe they had failed at a task, people with PMS/PMDD in the luteal phase described themselves more negatively and experienced more sadness and irritation.^39^ This effect did not present in people taking hormonal contraceptives, suggesting that endogenous hormonal cyclicity is involved.^39^ Thus, the present findings fit well with previous evidence of a negative bias in individuals with PMS/PMDD in the premenstrual phase and extend the findings to suggest an enhanced ability to detect sad emotions independent of current cycle phase. Given our preliminary evidence of enhanced ability to detect surprise during the premenstrual phase in PMDD, further research should examine the extent to which the PMDD-PEDA represents a general negativity bias or more of an FED advantage that also extends to positive emotions.

### Severity of PMDD associated with detection of disgust

Level of PMDD symptoms influenced the intensity and accuracy of detection for disgust emotions. The three PMDD level groups showed differences in disgust detection in terms of: (a) the intensity at detection (medium-large effect size) and (b) accuracy (large effect size). Interestingly, the mild PMDD group displayed the worst detection of disgust, being slower (i.e. required a higher intensity) than the moderate-severe PMDD group, and lower accuracy than both the no PMDD and moderate-severe PMDD groups. It should be noted that the subsample of people reporting moderate-severe PMDD was small (*n* = 8). When people reporting provisional PMDD were combined across symptom severity and compared to people without PMDD (i.e. no PMDD versus PMDD), the groups did not differ in intensity or accuracy for disgust (other than a non-significant trend for accuracy). When examining the full continuum of PMS/PMDD symptoms, the results do not point toward a linear relationship between PMDD symptoms and disgust detection. Furthermore, the most robust finding seems to be that, within individuals reporting provisional PMDD, mild symptoms are associated with less accurate and later detection of disgust, and moderate/severe symptoms are associated with more accurate and earlier detection of disgust. While replication with large sample prospective designs is required, these findings suggest a need to separately examine individuals with mild and moderate-severe PMDD when examining disgust detection. It is possible that combining these two groups into one PMDD group may obscure a dose-effect relationship within people with PMDD.

In considering possible explanations for the disgust findings, it is noteworthy that past research has linked the detection of disgust emotions to estrogen and progesterone levels. Some studies have found that high estrogen levels are associated with lower accuracy in detecting disgust emotions,^21,40^ while high progesterone is associated with better/lower intensity ratings for disgust emotions.^41^ One recent study suggested that individuals with PMDD exhibit lower estrogen and higher progesterone levels^42^ which might explain our finding of enhanced detection of disgust. The luteal phase is associated with a rapid rise in estrogen and progesterone, and then a rapid fall of both hormones in the premenstrual phase.^43^ These rapid hormone changes likely contribute to our finding that PMDD severity is associated with disgust detection. Similarly, people with menstrual cycles experience weaker feelings of disgust during high estrogen and low progesterone periods, such as around ovulation, which is proposed to be adaptive for mating behavior.^44^

Like the premenstrual phase, the immediate postpartum period is characterized by a rapid drop in estrogen and progesterone. In addition, many people experience postpartum depression (PPD), which is postulated to be partly caused by this hormonal change and has many overlapping symptoms with PMDD.^45^ Consistent with FED in depression, people with PPD are better at detecting negative emotions, and tend to view neutral infant facial expressions as more negative.^46^ This has been conceptualized as an adaptation toward perceiving their infant’s distress, consistent with the primary caretaker hypothesis, and the fitness threat hypothesis.^47–49^ Interestingly, high postpartum anxiety may be associated with perceiving disgust as more intense.^46^ However, another study found that PPD was associated with decreased accuracy in detecting disgust.^50^ The PPD findings may be relevant as the postpartum period and the premenstrual phase are both characterized by a similar steep decline in hormones, which may suggest similar mechanisms affecting disgust detection in PPD and PMDD.

Individuals who experience PPD or PMDD may have a hormonal sensitivity syndrome (HSS), and be differentially (and usually negatively) affected by hormonal changes.^51^ People with an HSS are more likely to have: PMS, higher rates of postpartum symptoms, and a history of antidepressant use. They also report lower sex drive, which is relevant in the context of decreased disgust processing around ovulation being adaptive for mating. Other studies have also found that PPD and PMDD symptoms tend to be experienced by the same people.^52,53^ The current disgust findings require replication, and more research is needed on the dimensional characteristics of PMDD (i.e. severity level) and disgust detection before a definitive conclusion can be drawn. However, the findings of enhanced premenstrual sadness and disgust detection for people with PMDD are noteworthy given a previous finding of enhanced working memory for only sad and disgust faces (not anger, fear, surprise, or happy) during the low (days 1–2) versus higher (days 4–13) hormone days of the menstrual cycle.^40^ Taken together, the findings suggest that detection of disgust and sad emotions is enhanced in individuals with PMDD during the premenstrual (and possibly early menstrual) phase, perhaps due to greater sensitivity to declining and low estradiol and progesterone levels.

### Limitations and strengths

The study has five main limitations. First, all groups were created based on self-report of PMDD symptoms without confirmation with 2-month prospective daily symptom ratings. While this may introduce retrospective bias in frequency and severity of PMDD symptoms, the measure of PMDD symptoms was based on *DSM*-5 criteria. Second, the sample for the moderate-severe PMDD group was small, and many large effect-size group differences were non-significant or trends. While this is a limitation, this is the first study to examine severity of PMDD symptoms with respect to FED, and yielded significant findings (i.e. intensity and accuracy for disgust). Future studies should aim for replication using larger sample sizes to increase power. Third, we utilized a between-subjects design. Replication with a within-subjects design is needed where participants are tested twice during their menstrual cycle (i.e. premenstrual and non-premenstrual phase). Fourth, cycle phase was determined using self-report as opposed to testing luteinizing hormone surges prospectively. Finally, while use of the FEDT allowed for measures of intensity and enhanced study power by including 15 responses per trial, future iterations of the task could increase the number of trials per emotion to increase the power of the individual emotion scores.

There were many strengths to the current study. One main strength relates to the design of the FEDT. The task was comprehensive in testing all six basic emotions using full face stimuli, and testing across different intensity levels. As emotion recognition abilities are more challenged in complex studies, this may have allowed for the detection of subtle group differences.^54^ We also calculated separate intensity and accuracy scores, allowing us to pinpoint which FED process differed between groups. Additional strengths pertain to our groups. Stringent exclusion criteria eliminated external confounds that may affect detection (e.g. taking hormonal medications/contraceptives in the past two months, recent alcohol consumption). Our overall sample size was also the largest of all the FED and PMS/PMDD studies.

## Conclusion

The present findings may help provide insight into the etiology of PMDD, some of the negative symptoms in PMDD, and potential adaptive value for PMDD. Past research has suggested that people with high PMS/PMDD symptoms may exhibit both a hormonal sensitivity, and trait-like negative cognitions. The results are consistent with a negativity bias, somewhat similar to what is seen in depression, as people reporting provisional PMDD showed enhanced processing of sad facial emotions, particularly when in the premenstrual phase. However, the advantages of enhanced emotion detection must also be considered and studied in PMDD. An association between PMDD severity and disgust detection was also found, which could provide a health and safety advantage. This was the first study to investigate intensity at detection in PMDD. The findings require replication, particularly since subgroup analyses had small samples, but they provide preliminary evidence of a PMDD-PEDA, supporting the concept of PMDD as a valid and unique depressive disorder.

The results point toward people with high PMS/PMDD symptoms differing from other menstruating individuals in emotion detection both across the cycle and within the premenstrual phase. General group differences in emotion detection are reflected by earlier detection of sad emotions by individuals with PMDD. Furthermore, people with PMDD may experience a hormonal sensitivity or hormonal mechanism that is activated within the normal hormonal milieu of the premenstrual/late-luteal phase and which contributes to more accurate detection of sad emotions (and possibly other emotions) at that time.

## Supplemental Material

sj-docx-1-whe-10.1177_17455057241259176 – Supplemental material for Symptoms of premenstrual dysphoric disorder and cycle phase are associated with enhanced facial emotion detection: An online cross-sectional study Supplemental material, sj-docx-1-whe-10.1177_17455057241259176 for Symptoms of premenstrual dysphoric disorder and cycle phase are associated with enhanced facial emotion detection: An online cross-sectional study by Bianca Boboc and Kirsten A Oinonen in Women’s Health

sj-docx-2-whe-10.1177_17455057241259176 – Supplemental material for Symptoms of premenstrual dysphoric disorder and cycle phase are associated with enhanced facial emotion detection: An online cross-sectional study Supplemental material, sj-docx-2-whe-10.1177_17455057241259176 for Symptoms of premenstrual dysphoric disorder and cycle phase are associated with enhanced facial emotion detection: An online cross-sectional study by Bianca Boboc and Kirsten A Oinonen in Women’s Health

## Declarations

**Ethics approval and consent to participate:** The study was conducted in accordance with the Declaration of Helsinki and approved by the Lakehead University Research Ethics Board (Ref No. 1469489). All participants provided written informed consent and received compensation (course credit and/or gift card draw) for participation.

**Consent for publication:** Written informed consent for publication was obtained from all participants.

**Availability of data and materials:** The data are available from the authors upon reasonable request.
