# Supplementary material for: Symptoms of premenstrual dysphoric disorder and cycle phase are associated with enhanced facial emotion detection: An online cross-sectional study
Source: Womens Health (Lond). 2024 Jun 14;20:17455057241259176. doi: 10.1177/17455057241259176 (PMC11179547; doi:10.1177/17455057241259176)
Supplement: sj-docx-2-whe-10.1177_17455057241259176 – Supplemental material for Symptoms of premenstrual dysphoric disorder and cycle phase are associated with enhanced facial emotion detection: An online cross-sectional study [file sj-docx-2-whe-10.1177_17455057241259176.docx]

**Supplementary Material**

**For: Symptoms of Premenstrual Dysphoric Disorder (PMDD) and Cycle Phase are Associated with Enhanced Facial Emotion Detection: An Online Cross-Sectional Study**

**Authors: Boboc, B., & Oinonen, K.A.**

**Journal: Women’s Health**

**Additional Information About the Facial Emotion Detection Task**

**Training Session**

The task commenced with a Training Session, to acquaint the participants with the mapping of responses on the keyboard. First participants were shown images with the seven facial emotions used in the task at 100% intensity and asked to respond to them using the appropriate keys. While each face image was presented, instructions relating to the mapping of response options on the keyboard also remained on the screen. Only once participants pressed the appropriate key response was the next image shown.

Participants were then told that they would be shown several more images of facial emotions, and to respond to them by pressing the appropriate key response as quickly as they can. Two images of each facial emotion type at 100% intensity (14 images total) were shown in

random order, and participant accuracy was measured. For each image, once participants indicated the emotion they perceived, only then was the next image presented.

**Practice Trial and Measurable Trials**

Following the training session to orientate participants to the Facial Emotions Task, they completed a Practice Trial identical to the task. Participants viewed an image of a neutral facial expression, that over 15 steps morphed into a distinct emotion, either anger, disgust, fear, happiness, sadness, or surprise. Participants were informed that all trials will commence with an image of an emotionally neutral face, which over the course of 15 images will morph into a detectable emotion, either anger, disgust, fear, happiness, sadness, or surprise. Participants were shown one image at a time and were instructed to indicate what emotion they perceived using the appropriate keys on the keyboard that were learned in the Training Session. The exact instructions participants were given are, “You will be shown an image of a neutral face which, over the span of 15 images, will gradually morph/change into one of the following emotions: disgust, fear, sad, angry, happy, or surprise. For every image in the morph indicate what emotion you see. We are interested in how fast and accurately you can identify the emotion. The first face of each morph will appear neutral. Begin by pressing the SPACE BAR to indicate this. Do this for all of the following faces that appear neutral. Once you see an emotion in the face, press the key corresponding to the emotion that you see. If you see an emotion but are unsure what it is, please only guess the emotion you see when you are reasonably confident. For each image try to respond as quickly as possible.”

For each image, once participants indicated the emotion they perceived, only then was the next image in the morph presented, and participants were required to respond to each image in the morph, regardless of whether or not they responded correctly. As in the Training Session, the mapping of the keyboard response options remained on the screen while each face image was presented. Following the Practice Trials, participants completed 24 trials of the Facial Emotions Task (four of each emotion). Each trial proceeded identically to the Practice Trial.

**Error Minimization**

Several measures were put in place to minimize errors on the task. First, participants were only permitted to respond with “neutral” for the first image of each morph. If participants attempted to give a different response, they were reminded of the task instructions. Secondly, trials in which participants appeared to give invalid responses were not included in analyses. Invalid responses included three patterns of responding: (a) participants did not change response options (i.e., persisting in responding *neutral* to all faces in the trial), (b) randomly oscillated between response options (i.e., if participants oscillated between responses at least four times in a row or if participants reported four or more emotions, not including neutral), and (c) incomplete trials (i.e., participants stopped responding halfway through the trials, or did not start the trial). Finally, responses were examined to identify any single key mistakes, where participants indicated an incongruent emotion for *one* image in the trial. The incongruent response had to follow at least two congruent responses (e.g., responding “happy” for at least two images, then randomly responding “sad” on one image, and then responding “happy” again for the subsequent image). In this case the singular incongruent response was considered a mistake and was modified to the emotion reported in the adjacent images. All trials were manually inspected for invalidity and single key mistakes by two raters.
